# The Effect of Projectiles on the Great Vessels

**Published:** 1918-09

**Authors:** 


***The Effect of Projectiles Allowed to Remain in Contact with the Walls of the Great Vessels.*** By René Le Fort. Report before the Académie de Médecine, June 11, 1918.

As frequently the elastic tissues of the vessel walls serve to stop a projectile at the end of its course, the author has sought to establish the effect of such contact by careful observation of a number of cases. He believes the following principle is borne out by his investigations. A projectile does not wear upon or perforate a vessel unless it is immovable before it.

Most projectiles so situated are small and easily moved, and hence are not injurious. They may, however, be so compressed against the walls of the vessel by external or muscular action as to cause serious friction against the rough edges of the missile.

The majority of vessel ulcerations due to the presence of foreign bodies are the result of infection. A bit of shell may carry dormant organisms for several years before they cause a serious infection. Such conditions are responsible for most cases of hemoptysis from unremoved foreign bodies.

The author enumerates the means of protection which prevent injury from the contact of missiles. The vessel and the missile are both encased in sclerous tissue which offers complete protection. The wall of the vessel in one instance was known to have developped in such a manner as to envelop a small shell fragment the size of a lentil.

Thrombosis is as rare as late hemorrhage. If a projectile pierces the wall of a vessel it results at once in a hemorrhage, in the healing of the vessel, or else in its complete obliteration.

The means of defense of vessels against injury are so effective that they should be taken seriously into account in judging the indications for extraction of foreign bodies. Pulsation of the body is not in itself an indication for extraction.

